# Heterogeneity and Decomposition Analysis of Manufacturing Carbon Dioxide Emissions in China’s Post-Industrial Innovative Megacity Shenzhen

**DOI:** 10.3390/ijerph192315529

**Published:** 2022-11-23

**Authors:** Shiming Liao, Dong Wang, Ting Ren, Xuemin Liu

**Affiliations:** 1 School of Economics and Management, Harbin Institute of Technology, Shenzhen 518055, China; 2 Shenzhen Humanities & Social Sciences Key Research Base for Carbon Emission Peaking and Carbon Neutral Technology, Policy, and Management, Harbin Institute of Technology, Shenzhen 518055, China; 3 HSBC Business School, Peking University, Shenzhen 518055, China; 4 School of Economics and Management, Dongguan University of Technology, Dongguan 523006, China

**Keywords:** carbon emissions, driving factors, LMDI, manufacturing, megacity Shenzhen

## Abstract

Effectively reducing manufacturing carbon dioxide (CO_2_) emissions is a vital strategy for China to curb its rapidly rising carbon footprint. Features of such a reduction in manufacturing include an increase in the share of high-tech manufacturing and optimization of the energy consumption structure. This study aims to analyze the case of Shenzhen, a unique post-industrial innovative manufacturing megacity, for its leading experience in China’s manufacturing transition. Disaggregated manufacturing emissions data of Shenzhen, including 27 sub-sectors in four categories, were collected, and driving factors were identified by the logarithmic mean Divisia index (LMDI) method. The results suggest that: (1) CO_2_ emissions from Shenzhen’s manufacturing show a phased difference between 2008–2012 and 2012–2020. CO_2_ emissions embodied in electricity consumption have increased by over 30% in the former period and have remained stable at a high level of over 90%. (2) Significant heterogeneity of CO_2_ emissions in various manufacturing sectors is revealed, with the largest emissions sources being factories that make communication equipment, computers, and other electronic equipment. (3) Lower carbon intensity is the primary factor in reducing CO_2_ emissions, while the economic activity effect of manufacturing possesses a stimulating impact. (4) The marginal impact of restructuring on CO_2_ emissions is insignificant since the manufacturing and energy structures of Shenzhen have been upgraded to a low carbon level. Therefore, strengthening the power saving management and improving the energy efficiency of the manufacturing, rather than optimizing the manufacturing and final energy structures, will be a necessary potential solution to the problem of how to reduce CO_2_ emissions in Shenzhen’s manufacturing.

## 1. Introduction

The national carbon peak and carbon neutrality goals (“Dual Carbon Target”) are the extensive, far-reaching systemic transition of China’s economy and society. With the profound impact of global climate change on the ecological, economic, and social development of human society, a global consensus has formed around the effective management of energy consumption and greenhouse gas (GHG) emissions. Therefore, controlling and reducing CO_2_ emissions has become a common goal for academia, industry, government and the international community. After rapid economic growth and a lifestyle evolution in recent decades, China has become the world’s largest CO_2_ emitter and the leading energy consumer [1]. In 2009, the Chinese government committed to lower CO_2_ emissions per unit of GDP (carbon intensity) by 40% to 45% from the 2005 level by 2020. The commitment was achieved in advance. Furthermore, China has also announced a target of reducing its carbon intensity by 60–65% from the 2005 level of reaching the peak of its CO_2_ emissions around 2030 [2] and of neutralizing its CO_2_ emissions by 2060. The carbon intensity indicators had been incorporated into the medium and long-term development plans of all provinces, municipalities, and autonomous regions in China. The total energy consumption targets and requirements were also included in the National 14th Five-Year Development Plan [3], which was gradually distributed to each city level by level.

At the forefront of China’s reform and opening up, Shenzhen was listed as the first low-carbon pilot city in China in 2010. It pioneered in the field of green and low-carbon development, with the promulgation of the “Long-term Plan for Low-carbon Development in Shenzhen (2011–2020)” [4]. In 2012, Shenzhen was identified as the first comprehensive demonstration city for energy-saving and emission-reduction fiscal policies in China, and the total amount of water, gasoline, and diesel sales decreased simultaneously for the first time. Moreover, in 2013, Shenzhen launched China’s first carbon trading market. Currently, Shenzhen is a megacity with the highest economic output per unit area, the most active innovation and entrepreneurship, the most well-developed manufacturing, and the largest population density in China. However, manufacturing is not only the engine of industrialization and the pillar industry of the national economy but also an energy-intensive and emissions-intensive industry. During 1995–2015, the CO_2_ emissions of China’s manufacturing industry increased by approximately 220%, accounting for nearly 58% of the total emissions of China [5]. In 2020, Shenzhen became the first city in China where the added value of industrial enterprises exceeded 900 billion yuan, reaching 952.812 billion yuan, with an increase of 1.5%. The energy intensity of industry (energy consumption per unit of industrial GDP) was about 0.161 TCE per ten thousand yuan at the 2020 price, which was only 15.61% of the national level. Therefore, studying the CO_2_ emissions of manufacturing in Shenzhen has important practical significance for future manufacturing emission reduction and China’s CO_2_ emissions peak.

This paper analyzes the trend of CO_2_ emissions from Shenzhen’s manufacturing during 2008–2020 with the application of the LMDI method. The main contributions of this paper are fourfold: (1) Taking China’s megacity Shenzhen, the most representative post-industrial and innovative megacity, as the research object, and the impact of four factors on its manufacturing emissions is decomposed in detail. Although many scholars have studied the manufacturing emission drivers of China and its provincial regions, Shenzhen, as a national innovation city, has not received much attention regarding the evolution of its manufacturing emissions and the driving factors behind the trend. (2) An in-depth and systematic analysis of the internal structural changes in Shenzhen’s emissions from manufacturing was conducted, using detailed data of manufacturing sub-sectors in Shenzhen rather than aggregated data of all manufacturing industries. (3) Not only factors such as manufacturing structure and carbon intensity are analyzed, but also the energy structure of manufacturing emissions is decomposed, including direct emissions of fossil energy and indirect emissions of electricity, and comparisons between different periods are investigated. (4) The important policy implications of reducing CO_2_ emissions in Shenzhen’s future manufacturing industry are put forward, which will help promote energy conservation and technological innovation, establish a green manufacturing growth mechanism, and have a demonstration effect on the acceleration of high-quality development of manufacturing in China’s megacities.

The structure of the article is as follows: Section 2 provides a literature review; Section 3 introduces the methodology; Section 4 presents the results and discussions; while Section 5 discusses the conclusions and implications.

## 2. Literature Review

### 2.1. Methods for the Decomposition Analysis

Structural decomposition analysis (SDA), production–theoretical decomposition analysis (PDA), and index decomposition analysis (IDA) are three methods mostly used in decomposition analysis of the influencing factors [6]. The SDA method is a kind of decomposition analysis based on input–output tables [7]. However, the input–output tables are usually lagging and discontinuous, and there is a lack of input–output tables data in many regions, which limits its wide-scale use. Based on production theory, the PDA method has been gradually applied in the research area of environment and energy after its first presentation by Zhou and Ang [8]. Although some production technology-related components are included in PDA, it cannot give structural effects and its decomposition results can be affected by the number of entities considered. In terms of implementation, the use of PDA to solve the problems associated with linear programming is more complicated than the use of IDA/SDA with algebraic operations. One of the main reasons for choosing the IDA method is that its implementation process is relatively straightforward, the requirements for data in IDA are not high, and its results can be compared with previous studies. With the improvement of IDA, it can overcome the incomplete decomposition defects of the previous decomposition methods and can effectively solve the problem of zero value and negative value of data summary [9]. The biggest advantage of this method is that it can trace the cause of the change of dependent variables and determine the deep factors through the decomposition of subindustries, to provide the basis for the formulation of practical and reliable policy measures. This method has been widely used in the fields of energy economics and environmental economics. However, the IDA method has various sub-methods. Ang has provided a detailed analysis and recommends the use of LMDI-I [10]. Since it has no residuals, this method is easy to use, and the results are easy to interpret. This article uses the method recommended by Ang [10]. LMDI-I has two kinds of decomposition forms, namely additive form and multiplicative form. The only difference between them is the form of representation. To make it easier to understand, this article uses the additive form of LMDI-I.

### 2.2. Driving Factors of CO_2_ Emission Change

Representative studies about CO_2_ emission decomposition of the LMDI method are summarized in Table 1. These four factors, namely total production activity, industrial structure, energy structure, and energy/carbon intensity, have been the focus of existing studies. The majority of the literature about manufacturing CO_2_ emissions focuses on developed European countries. For example, Akbostanci et al. have revealed that coal is the decisive factor and that the iron and steel industry is the most polluting portion of Turkey’s manufacturing sector [11]. Hammond and Norman have found that the non-energy-intensive subsector of the UK’s manufacturing sector has a greater reduction than the energy-intensive subsector because of the greater improvement in energy intensity [12]. Jeong and Kim decomposed the GHG emissions of the UK’s manufacturing sector and found that the structure and intensity effects play roles in it [13]. In the developing world, Román et al. have shown that the key drivers of emissions in Colombia during 1990–2012 were the effects of wealth and population increase, and that the improvement in energy efficiency failed to offset the increasing effect of other factors [14]. Mousavi et al. have indicated that the major driver of Iran’s CO_2_ emissions is its increased consumption, and that the effect of technology-related improvements is very weak [15]. At present, China’s CO_2_ emissions have attracted the attention of many researchers, but most of them have focused on the national or provincial level. Liu et al. have shown that, in China, the ferrous metal smelting and rolling industry is the largest source of CO_2_ emissions, followed by chemical raw materials and products and then non-metallic minerals [5]. Wang and Feng have found that the secondary industry accounts for approximately 80% of total CO_2_ emissions with provincial panel data of three sectors in China [16]; however, the industrial structure effect and energy structure effect varied considerably over the years without showing clear trends [16]. Wu et al. found that, in Inner Mongolia, China, energy-intensive industries, such as energy production and processing, are the main sources of industrial emissions, and coal is the most important source of energy in the energy mix [17]. Wang et al. have indicated that the energy intensity effect plays a dominant role in promoting the decoupling status between CO_2_ emissions and industrial growth in Taiwan of China [18]. Feng et al. have shown that economic and demographic growth has positive effects on the CO_2_ emissions of Guangdong province [19]. The existing studies about manufacturing emissions mostly conducted index decomposition analysis at the national level or the provincial level. Only a few studies have investigated the emissions at the city level [20,21,22] and have focused on Beijing, Shanghai, Tianjin, Chongqing, and Zhuhai in China [23,24]. Gu et al. studied the determinants of Shanghai’s CO_2_ emission change during the period 1995–2015 and identified that energy intensity is the main factor for carbon mitigation, followed by economic structure, residential energy intensity, and emission coefficients [25]. Liu et al. also studied Shanghai’s experience of realizing CO_2_ emission stagnation during the period 2007–2012 [26]. Kang et al. found that both economic growth and industrial structure change increase CO_2_ emissions [27]. Tan et al. analyzed the driving factors of CO_2_ emission in Chongqing and predicted the future reduction potential [28]. Feng et al. explored the driving forces of industrial emissions in the coastal city of Zhuhai in China during the period 2006–2016 [24].

In summary, we find that a large number of existing studies have the following deficiencies. First of all, in terms of the research area, most of the current related literature is focused on the national or inter-provincial level of China, or some heavy industrial cities, but rarely study innovative megacities such as Shenzhen. Shenzhen uses less fossil energy but more electricity to generate and create more economic output, and has not received enough research attention. Second, in terms of the research industry, most studies on the energy structure and emission intensity are concentrated on the thermal power generation industry, the transportation industry, or the whole economic sector. Furthermore, those city-level studies only considered the aggregate-level emissions when performing the decomposition analysis and none of them studied the inner structure of manufacturing in an innovative megacity. It is necessary and important to study the decomposing analysis with disaggregated manufacturing data. Third, many empirical studies on the drivers of industrial CO_2_ emissions only consider direct CO_2_ emissions from the consumption of fixed energy sources (e.g., coal, oil, fuel oil, diesel, gasoline, natural gas, etc.), without taking into account indirect emissions from electricity use [29]. This means that the actual CO_2_ emissions may have been greatly underestimated. Fourth, many previous studies have neglected the decomposition of different stages. Consistent results about the emission drivers have also not been achieved yet. It is necessary and important to decompose the drivers of CO_2_ emissions by different stages according to the emission characteristics of manufacturing in the megacity.

## 3. Method and Data

### 3.1. Quantification of Carbon Dioxide Emissions

According to Shenzhen’s statistical yearbook, raw coal, crude, gasoline, kerosene, diesel oil, fuel oil, liquid petrol gas (LPG), natural gas and electricity are the nine major energies used in the manufacturing industry in Shenzhen. Energy activities account for more than 95% of Shenzhen’s GHG emissions [30]. Therefore, almost all sources of CO_2_ emissions from manufacturing are covered in this study. The applied method of CO_2_ emission quantification followed the guidelines of the Intergovernmental Panel on Climate Change (IPCC) [31] and the Guidelines for Provincial Inventory of Greenhouse Gas Emissions. The CO_2_ emissions arising from fossil energy combustion were calculated by the parameters of their energy calorific value and carbon emission factor [5,29,32]. Referring to other researchers [5,28], we calculate the CO_2_ emissions coefficient of each form of fossil energy, which is a product of net calorific value, carbon content, oxidation rate, and 44/12. It is then directly multiplied by the amount of physical energy consumption to get the direct CO_2_ emissions. In addition to the CO_2_ emissions from the combustion of fossil energy, this paper also considers the CO_2_ emissions embodied in the electricity consumption of manufacturing. We calculate the CO_2_ emissions of each kind of energy in each sector and then add them together to obtain the total emissions of each sector in manufacturing. The formula we use to calculate CO_2_ emissions of each manufacturing sector is shown in Formula (1).

(1) $$C_{j}=\sum_{i=1}^{9}C_{ij}=\sum_{i=1}^{8}AD_{ij}\times LCV_{i}\times CF_{i}\times O_{i}\times \frac{44}{12}+AD_{9j}\times f_{9}=\sum_{i=1}^{9}AD_{ij}\times f_{i},$$

where:

*i:* the type of energy from 1 to 9 (See Table 2)

*j*: the sub-sector of manufacturing from 1 to 27

*C_j_*: CO_2_ emissions of sector j in the manufacturing sector

*AD_i j_*: the physical amount of consumption of the *i* type of energy in sector *j* of manufacturing

*C_ij_*: the CO_2_ emissions of the *i* type of energy in sector *j* of manufacturing

*LCV_i_*: the lower calorific value of the *i* type of energy

*CF_i_*: the carbon content of the *i* type of energy

*O_i_*: the oxidation rate of the *i* type of energy

*f_i_*: the CO_2_ emission coefficient of the *i* type of energy

44/12: the relative atomic mass of CO_2_ and C

Generally, the CO_2_ emission coefficient of fossil energy combustion will not change significantly in a short period of time. The calculated CO_2_ emission coefficient for various energy sources in this study are shown in Table 2. *LCV_i_*, *CF_i_*, and *O_i_* are from the Guidelines for Provincial Greenhouse Gas Inventory (provided by the National Development and Reform Commission of China) and the appendix of the “China Energy Statistical Yearbook 2018”. The CO_2_ emission coefficients of main fossil energy from IPCC [31] are also presented in Table 2. Since the electricity of Shenzhen is mainly provided by China Southern Power Grid Corporation, the emission coefficient of the electricity of China Southern Power Grid is based on the report of the regional grid baseline emission factor and the ratio of non-fossil energy. The thermal power carbon emission coefficient reported in 2012 is about 0.9344 tons CO_2_/MWh [33], and the non-fossil energy of the Southern Power Grid accounts for 44% of the total generated electricity [34]. Then, the final calculated emission coefficient of electricity use is about 0.5233 tons CO_2_/MWh. This paper assumes that the CO_2_ emission coefficient for electricity remains the same for different years.

### 3.2. LMDI Method

Ehrlich and Holdren put forward the famous IPAT identity, a formula that states that environmental pressure comes from population size, per capita wealth, and the level of technology related to the environment [35]. Waggoner and Ausubel further decomposed the technology level T as the product of technology per unit of GDP and the environmental impact per unit of technology [36]. Kaya proposed the Kaya identity, a formula which links the factors of economy, policy, and population to CO_2_ emissions from human activities [37]. Grossman and Krueger proposed that economic growth affects environmental quality through scale, technology, and composition effects [38]. Manufacturing activities also affect industrial CO_2_ emissions mainly through scale, structure, and technology effects. First, the expansion of manufacturing activity increases the use of energy and then increases the amount of CO_2_ emissions if the nature of manufacturing activity remains unchanged. Second, different energies produce the same power with different CO_2_ emissions, and different industries produce the same economic output with different CO_2_ emissions. Therefore, the adjustment of the energy structure and the optimization of the industrial structure tend to reduce the proportion of carbon-intensive energy and polluting industries, while the proportion of clean industries and energy increases, resulting in the reduction of emissions. Finally, modern manufacturing technologies are typically cleaner than older technologies because of the growing awareness of CO_2_ emission reduction and energy conservation in production.

Referring to Ren et al. [29], Gu et al. [25], and Tu et al. [20], we use the LMDI method to identify the driving factors of the total CO_2_ emissions from Shenzhen’s manufacturing sector as follows:

(2) $$C_{mfr.}=\sum_{j=1}^{27}C_{j}=\sum_{j=1}^{27}\sum_{i=1}^{9}C_{ij}=\sum_{j=1}^{27}\sum_{i=1}^{9}\frac{C_{ij}}{C_{j}}\times \frac{C_{j}}{Y_{j}}\times \frac{Y_{j}}{Y}\times Y$$

where:

*C_mfr_*.: the total CO_2_ emissions of manufacturing

*Y_j_*: the added value of the industry *j* of manufacturing

*Y*: the gross domestic product of manufacturing, with 2010 as the base year

other variables are the same as the Formula (1).

Among Equation (2), the corresponding multipliers represent different influencing factors. The meanings and abbreviations of each multiplier are shown in Table 3 as follows. Based on Equation (2), we divide the factors affecting CO_2_ emissions of Shenzhen’s manufacturing into the following four categories: the emission structure of different energies (CS), CO_2_ emission intensity (CI), the added value structure of manufacturing (MS) and economic growth of manufacturing (Y). The CO_2_ intensity uses the ratio of the CO_2_ emissions to the added value of the manufacturing. It represents the level of CO_2_ emission efficiency by different sectors. The lower the CO_2_ emission intensity, the better the carbon-reducing technology in manufacturing industries and vice versa. The manufacturing structure adopts the ratio of the added value of each manufacturing industry to the total added value of manufacturing. The added value of manufacturing is the level of economic activity of manufacturing. The higher the economic activity, the higher the CO_2_ emissions. All zeros in the data set may be replaced by a small positive constant [9].

Therefore, according to the above definition, we rewrite Equation (2) as follows:

(3) $$C_{mfr.}=\sum_{j=1}^{27}\sum_{i=1}^{9}CS_{ij}\times CI_{j}\times MS_{j}\times Y$$

Based on Equation (3), we use the additive form of the LMDI method to decompose the change in CO_2_ emissions of manufacturing into four corresponding factors between the base year and the T year as follows:

(4) $$\Delta C_{mfr.}=C_{mfr.}^{T}-C_{mfr.}^{0}=\Delta C_{CS}+\Delta C_{CI}+\Delta C_{MS}+\Delta C_{Y}$$

The emission energy structure effect ($\Delta C_{CS}$) means the impact of changes in the structure of different energies on the total CO_2_ emissions. The CO_2_ emission intensity effect ($\Delta C_{CI}$) can reflect changes in CO_2_ emissions due to increased emission efficiency, such as low-carbon technologies. The manufacturing structure effect ($\Delta C_{MS}$) is the impact of changes in the added value proportion of each sector on CO_2_ emissions, usually indicating a rise in high-tech manufacturing. The economic activity effect of manufacturing ($\Delta C_{Y}$) indicates that changes of the manufacturing economy affect the changes in CO_2_ emissions. The total of each effect is then equal to the change in total CO_2_ emissions from manufacturing. The corresponding effects are calculated as follows:

(5) $$\Delta C_{CS}=\sum_{j=1}^{27}\sum_{i=1}^{9}W_{ij}\cdot \ln\left(\frac{CS_{ij}^{T}}{CS_{ij}^{0}}\right)$$

(6) $$\Delta C_{CI}=\sum_{j=1}^{27}\sum_{i=1}^{9}W_{ij}\cdot \ln\left(\frac{CI_{i}^{T}}{CI_{i}^{0}}\right)$$

(7) $$\Delta C_{MS}=\sum_{j=1}^{27}\sum_{i=1}^{9}W_{ij}\cdot \ln\left(\frac{MS_{i}^{T}}{MS_{i}^{0}}\right)$$

(8) $$\Delta C_{Y}=\sum_{j=1}^{27}\sum_{i=1}^{9}W_{ij}\cdot \ln\left(\frac{Y^{T}}{Y^{0}}\right)$$

Among these,$\;W_{ij}=\{\begin{array}{c} \frac{C_{ij}^{T}-C_{ij}^{0}}{\ln\left(C_{ij}^{T}/C_{ij}^{0}\right)},\;C_{ij}^{T}\cdot C_{ij}^{T}\;\neq 0\;\\ C_{ij}^{T},\;C_{ij}^{T}=C_{ij}^{T}\;\\ 0,\;C_{ij}^{T}\cdot C_{ij}^{T}=0\; \end{array}$ represents the average logarithmic weight.

### 3.3. Data Sources

Because of the data limit, our research period is from 2008 to 2020. The energy activity data of different manufacturing sectors are from the Shenzhen Statistical Yearbook 2009–2021 published by the Shenzhen Statistical Bureau [39]. The total added value data of the industry is also from the Shenzhen Statistical Yearbook 2021. All value-added data are at the 2010 price based on the industrial value-added index. In this study, we used the structure of the added value of the above-sized manufacturing. The added value data of above-sized manufacturing by different sectors during 2008–2020 in Shenzhen come from the Guangdong Statistical Yearbook 2009–2021. The added value of industry above the designated size accounts for about 90% of the total industrial added value. There are very small changes in the definition of manufacturing classification after 2013. For the format consistency of manufacturing data in different years, we divide Shenzhen’s whole manufacturing economy into 27 sectors and 4 categories, as shown in Table 4.

## 4. Results and Discussions

### 4.1. Characteristics of CO_2_ Emissions from Shenzhen’s Manufacturing

**Figure 1:**
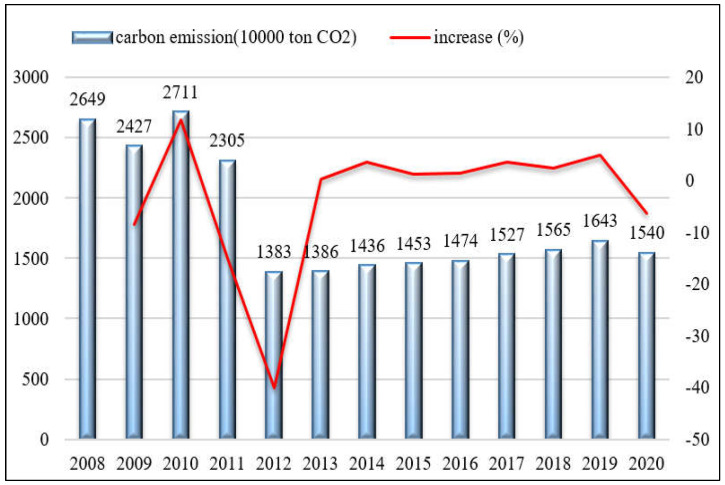
CO_2_ emissions of Shenzhen’s manufacturing from 2008 to 2020.

**Figure 2:**
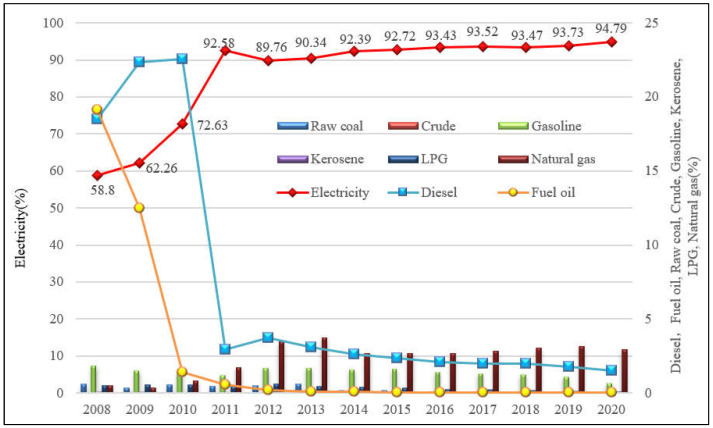
CO_2_ emissions proportions of various energy sources in Shenzhen’s manufacturing.

(1) Overview of total CO_2_ emissions from manufacturing. Figure 1 illustrates the total CO_2_ emissions from Shenzhen’s manufacturing from 2008 to 2020. CO_2_ emissions from Shenzhen’s manufacturing in 2020 are only 58% of those in 2008. This change is equivalent to an average annual reduction rate of 4.42%. However, there is a significant difference between the emission rate from 2008 to 2012 and that from 2012 to 2020. The rapid decline of CO_2_ emissions during 2008–2012 is because, since the global financial crisis in 2008, Shenzhen has been actively pushing for industrial transition and upgrading, especially the manufacturing. Another reason is Shenzhen’s early attention to the air pollution problem of megacities in China [30] and the rapid electrification of its manufacturing. Since CO_2_ emission control and air pollution control are highly rooted in the same origin in China, the reduction of CO_2_ emissions is widely regarded as one of the synergies of air pollution control [22,30]. As a consequence, low-end industries with high fossil energy consumption and pollution are being quickly phased out. Since 2012, Shenzhen has been continuously introducing policies to support strategic emerging industries, forming several major industries such as the internet, cultural creative, new energy, and new materials. CO_2_ emissions have since reached 15.40 million tons in 2020 with a small, negligible increase each year. The reason for the subtle increase during 2012–2020 is mainly due to the rapid development of high-tech manufacturing industries such as electronic information and precision manufacturing.
(2) CO_2_ emissions under the energy structure. Figure 2 shows the energy structure of CO_2_ emissions from Shenzhen’s manufacturing during the period 2008–2020. Electricity is obviously the primary source of emissions in Shenzhen’s manufacturing, showing a completely opposite development trend to oil and gasoline. Specifically, the embodied CO_2_ emissions of electricity accounted for nearly 60% of total emissions in 2008 and increased to 95% in 2020. After 2011, its proportion exceeded 90% of total manufacturing CO_2_ emissions. The sum of the CO_2_ emissions proportion of diesel and fuel oil was 37.6%, 34.8%, and 24.0% in 2008, 2009, and 2010, respectively. After 2011, the CO_2_ emission proportion of the two energies fell to a range of between 2% and 4%. The proportion of electricity in Shenzhen’s manufacturing stays at a relatively high level. Electrification reduces direct CO_2_ emissions and air pollutants and improves the living environment. The improvement in air quality will have significant impacts on health benefits [40]. This may be one of the reasons why Shenzhen has attracted a large number of high-tech talents, and the inflow of talents has helped its rapid development in high-tech industries.

### 4.2. Heterogeneity of CO_2_ Emissions from Shenzhen’s Manufacturing

**Figure 3:**
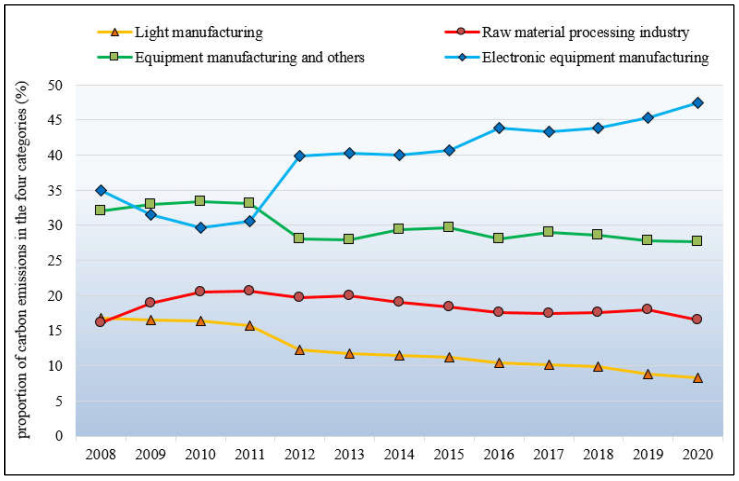
Industry heterogeneity of CO_2_ emissions from manufacturing in Shenzhen.

(1) Industry heterogeneity. Figure 3 shows the industry heterogeneity of CO_2_ emissions from manufacturing in Shenzhen during 2008–2020. According to industry characteristics, 27 sub-sectors are divided into four categories, revealing the sector structure of CO_2_ emissions from Shenzhen’s manufacturing. Specifically, electronic equipment manufacturing is the primary sector responsible for energy emissions, accounting for nearly 34.93% of total manufacturing emissions in 2008 and increasing to 47.43% in 2020. Before 2012, the proportion of its emissions was slightly reduced to about 30% but has gradually increased since. The emission proportion of light industry to the total proportion of manufacturing has dropped from 16.86% in 2008 to 8.26% in 2020. During the period under study, the emission share of equipment manufacturing and others in manufacturing decreased from 32.1% to 27.73%. The emission proportion of the raw material processing industry increased slightly and then decreased slightly, and the proportion in 2020 (16.57%) and 2008 (16.11%) is basically the same.
(2) Electricity heterogeneity. Table 5 shows the proportion of CO_2_ emissions embodied in electricity to the total CO_2_ emissions in individual subsectors during 2008–2020. After 2011, their values increased significantly. This is the same conclusion obtained in Figure 2. In 2020, the proportion of CO_2_ emissions embodied in electricity from the smelting and pressing of ferrous metals (Sector ID_18), processing of petroleum, coking, and nuclear fuel (Sector ID_12), and manufacturing of wine, beverages, and refined tea (Sector ID_3) is around 65%. The proportion of CO_2_ emissions contained in electricity in other individual industries is above 70%. There are more than 50% of subsectors whose electricity-triggered CO_2_ emissions occupied over 90% of their total emissions. Among these, three subsectors (sector ID_27, ID_24, and ID_16) reach as high as 95%. Therefore, in Shenzhen, a city in the post-industrial era, indirect CO_2_ emissions triggered by electricity usage are the most important emission source in its manufacturing industries.

An important feature of the emission evolution in Shenzhen’s manufacturing is the shift from direct emissions from high-emitting heavy industries to indirect emissions from high-tech manufacturing industries. At present, high-tech manufacturing is widely regarded as a low-carbon industry, which can reduce emissions by replacing heavy industry [29,41]. However, attention should be paid to indirect emissions in the supply chain of high-tech manufacturing [26,42]. This paper shows more specifically that high-tech manufacturing industries have low direct emissions from fossil energy combustion, but they may have more indirect emissions through the massive consumption of electricity.

### 4.3. Decomposition Analysis of CO_2_ Emissions in Shenzhen’s Manufacturing

**Figure 4:**
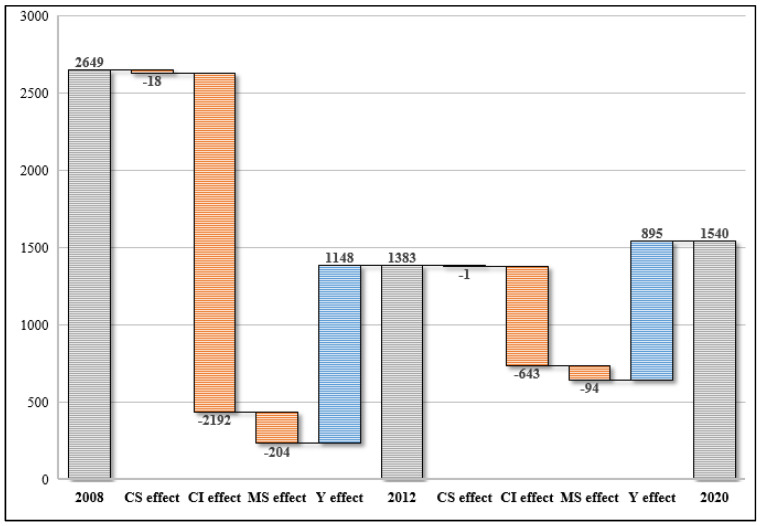
Decomposition results of various driving forces to CO_2_ emissions changes in Shenzhen’s manufacturing.

(1) Decomposition of different driving factors. The decomposition of CO_2_ emissions changes and the contributions of various driving factors from 2008 to 2020 are shown in Table 6. The contributions of various driving forces refer to the proportion of CO_2_ emissions changes caused by each factor. Each factor presents a different effect in the previous year. During the period 2008–2020, the overall CO_2_ emission in Shenzhen’s manufacturing decreased by 41.87%. CO_2_ emission intensity decreased by 107.04%, followed by manufacturing structure (−11.24%) and energy emission structure (−0.71%). Manufacturing economic activity emissions increased by 77.13% and is the only positive driver. The period from 2008 to 2012 was the most important structural adjustment stage for Shenzhen’s manufacturing CO_2_ emission changes. It can be seen that the reduction of manufacturing emission intensity is the most important factor for the decrease [18,19,24]. All along, Shenzhen has taken the strategy of a high-quality economy as a long-term strategy for urban development, strived to become an important role in global innovation and development, and treated innovation as its primary driving force for sustainable urban growth. Shenzhen vigorously lays out seven “strategic emerging industries” (next-generation information technology industry, high-end equipment manufacturing industry, green low-carbon industry, biomedical industry, digital Economy Industry, new materials industry, and marine economy industry) and four “pillar industries” (cultural and creative industries, high-tech industry, modern logistics, and financial industry), promotes the transformation of the economy into high-tech industries, strives to improve the quality of economic development, and continuously enhances the added value of the industry. The high-quality development of the manufacturing industry is the top priority of Shenzhen’s industrial development strategy. In 2020, the added value of Shenzhen’s high-tech manufacturing and advanced manufacturing accounted for 66.1% and 72.5% of the added value of industrial enterprises above designated size, respectively [43]. The development of high-tech, high-value-added, low-carbon industries is an important reason for the decline in manufacturing emissions [26]. Therefore, it can be seen that there are two significantly different stages in the change of manufacturing CO_2_ emissions in Shenzhen, and the driving factors are also different in those two stages.
(2) Decomposition at different stages. Since there is a clear trend difference between the two stages of 2008–2012 and 2012–2020 in Shenzhen’s manufacturing CO_2_ emissions, it is necessary to examine the impact of different factors on these two periods. From Figure 4, each factor presents a different effect in different stages. Overall, the manufacturing activity effect ($\Delta C_{Y}$) remains positive during these two periods, which is the primary driving force of the changes in emission. However, the carbon emission intensity effect ($\Delta C_{CI}$) is the key factor that offsets the increase of manufacturing emissions at the two stages. In addition, the energy structure effect ($\Delta C_{CS}$) also contributes a small decrease in emissions during 2008–2012 but changes to nearly none during 2012–2020. Relative to the carbon intensity effect, the energy structure effect is insignificant. The manufacturing structure effect ($\Delta C_{MS}$) is negative in both stages. From 2008 to 2012, the key factor for the decrease in CO_2_ emissions was carbon intensity, which decreases 21.92 million tons of emissions accumulatively in Shenzhen’s manufacturing sectors. After the financial crisis in 2008, external demand slowed down and costs increased. Shenzhen firmly grasped the mechanism of the international financial crisis and took the initiative of industrial upgrading. With the help of the market, Shenzhen phased out of backward production capacity and released part of the industrial land to make room for the development of newly emerging industries. At the same time, it actively deployed strategic emerging industries and improved the industrial level, covering a wide range of high-tech fields such as electronic information, aviation, and microchips. From 2012 to 2020, the key driving factor of CO_2_ emissions changes to manufacturing activity, which caused 8.95 million tons of emissions in Shenzhen’s manufacturing sectors. In fact, CO_2_ emissions are closely linked to the growth of GDP [24]. In general, countries with larger economic output have larger CO_2_ emissions, and vice versa [44]. In addition to the impact of total economic activity, CO_2_ emissions are also closely related to the industrial structure and emission intensity. The emission intensity reduction effect offset 6.43 million tons of CO_2_ emissions, and industrial structure changes offset 0.94 million tons of emissions. However, the incremental effect of manufacturing activities exceeds the deductive effects of other factors. In recent years, Shenzhen has accelerated industrial transition and upgrading processes, and used market instruments, such as CO_2_ emissions allowance trading, to promote the reduction of emissions among its manufacturing companies. The annual emission compliance rate of Shenzhen’s CO_2_ emission control units has been relatively high. From 2013 to 2020, the compliance rates of enterprises included in the CO_2_ emissions allowance trading market were over 99.0% each year. Therefore, as a post-industrial and innovative megacity, Shenzhen first experienced a stage in which low-end and backward industries were eliminated due to the rapid transition and upgrading of the manufacturing industry, manifesting as a rapid reduction in CO_2_ emissions (2008–2012). Then, with the rapid development and growth of various strategic emerging industries and innovative technology industries with low carbon intensity, the second stage of a slow increase in carbon emissions was formed (2012–2020). The optimization of industrial structure can reduce local CO_2_ emissions or pollution [26] but can cause the transferal of emissions and pollution to other places [21,22]. One of the most important ways for a megacity to transfer direct CO_2_ emissions is to import large amounts of electricity [26,45]. The essence of this is the relocation of the fossil energy generation industry.

### 4.4. Changes of CO_2_ Emissions Intensity and Manufacturing Structure

**Figure 5:**
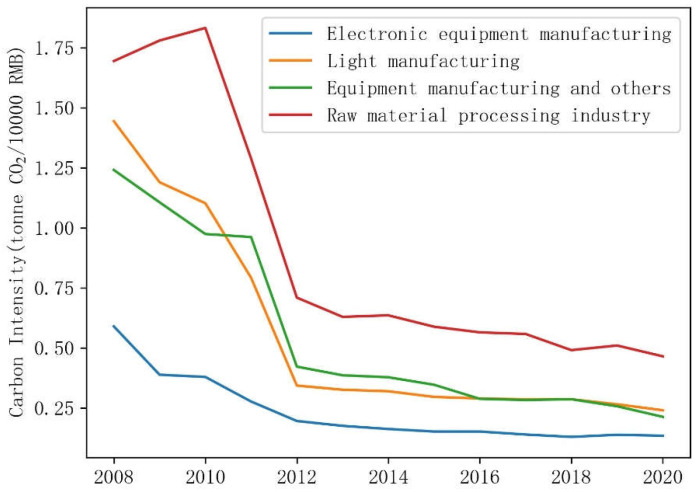
Trend of carbon intensity of Shenzhen’s manufacturing sector by four categories during 2008–2020.

**Figure 6:**
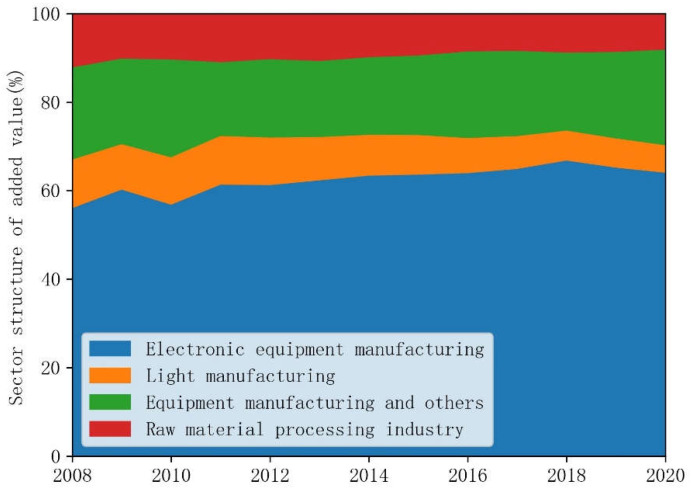
Proportion trend of added value of Shenzhen’s manufacturing sector by four categories during 2008–2020.

(1) Evolution of carbon intensity. Figure 5 shows the trends of CO_2_ emissions intensity of Shenzhen’s manufacturing by four categories during 2008–2020. We can see that all four of the categories’ CO_2_ emission intensities declined by more than 2/3 during these years. CO_2_ intensity represents the total amount of CO_2_ emissions per unit of GDP, and its reciprocal is CO_2_ productivity, which reflects the efficiency of CO_2_ emissions. As early as 2010, Shenzhen was listed as one of the first low-carbon pilot areas. In 2012, Shenzhen planned the CO_2_ emission allowance trading market. In 2013, Shenzhen’s emission allowance trading system was officially launched. The first batch of 621 manufacturing enterprises was included. These manufacturing enterprises are decreasing their emissions while increasing industrial value added. Therefore, the sharp decline in the CO_2_ intensity of the Shenzhen manufacturing industry is likely to indicate that the emission efficiency of the sub-sectors has been significantly improved. Efficiency improvement is an important way for cities to slow the increase in their CO_2_ emissions [21]. The manufacturing technological level is improved, and the potential for emission reduction is fully released. In addition to the changing trends of CO_2_ emission intensity, Figure 5 also shows the difference in CO_2_ intensity between different industries. Electronic equipment manufacturing with the highest CO_2_ emissions has the lowest CO_2_ intensity among these four categories of manufacturing. Manufacturing industries that process raw materials have the highest carbon intensity. While light industry and equipment manufacturing and others are in the middle level. The deep transition of the manufacturing industry is also one of the bases for the peak of total CO_2_ emissions [26]. Therefore, the industries with the lowest CO_2_ emission intensity are already the most important sectors in Shenzhen’s manufacturing, which means that the potential for emission reduction by lowering its CO_2_ intensity is exhausted unless Shenzhen increases the share of electricity generated from renewable sources and the efficiency with which it is used.
(2) Trend in manufacturing value added. Optimizing industrial structure is an important way to promote direct CO_2_ emission reduction and pollution control [22,28]. Figure 6 shows the proportion trend of the added value of Shenzhen’s manufacturing sector by four categories during 2008–2020. The proportion of the added value of electronic equipment manufacturing in the total added value of the manufacturing increased from 55.71% in 2008 to about 63.72% in 2020. According to the National Economic Industry Classification Standard published by the National Bureau of Statistics in 2017 [46], manufacturing of communication equipment, computers, and other electronic equipment (manufacturing sector ID_27) includes computer manufacturing, communication equipment manufacturing, radio and television equipment manufacturing, radar and ancillary equipment manufacturing, non-professional audiovisual equipment manufacturing, manufacturing of smart consumer devices, manufacturing of electronic devices, manufacturing of electronic components and electronic materials, and manufacturing of other electronic devices. This industry has relatively high technological intensiveness and industrial added value with low carbon intensity, and plays an irreplaceable and fundamental role in improving both the industrial economy and the quality of life of ordinary citizens. The second largest added value share is equipment manufacturing and others. Its proportion of added value in the total added value of manufacturing is around 20%, which is a slight increase of about 1% in 2020 compared to its value in 2008. The value added share of light manufacturing decreased from 11% in 2008 to 6.22% in 2020. The proportion of the raw material processing industry decreased from 12.47% in 2008 to 8.45% in 2020. The process of optimizing the industrial structure of a city with a strict environmental policy can lead to the transfer of pollution and heavy industry to other cities. Collaborative control of CO_2_ emissions and pollution with neighboring regions is also important [21,22,30]. The impact of Shenzhen’s industrial relocation on CO_2_ emissions on other surrounding cities needs to be deeply analyzed to reduce carbon leakage in the future.

## 5. Conclusions and Implications

In order to analyze the current situation of CO_2_ emissions from manufacturing subsectors with four categories in Shenzhen during 2008–2020 and the driving factors behind these changes, this paper used CO_2_ emission coefficients to calculate the CO_2_ emissions of different energy types in various industries and conduct structural analysis. The LMDI method was introduced to decompose and analyze the impact of manufacturing economic activities, manufacturing structure, emission intensity, and energy structure of emissions on the changes in its total manufacturing emissions. Based on the above findings and discussion, we make the following conclusions:

First, the successful transition and upgrading of the industry led to a cliff-like decline in the total CO_2_ emissions of Shenzhen’s manufacturing industry from 2008 to 2012, which has been well maintained and controlled since then. Furthermore, from the perspective of energy structure, electricity is the main energy source of emissions in Shenzhen’s manufacturing industry with a continuous and stable trend, followed by diesel and then fuel oil, whose proportion has dropped sharply since 2011. That is, the CO_2_ emissions from the direct use of fossil energy in manufacturing enterprises in Shenzhen account for a very low proportion of total CO_2_ manufacturing emissions, but the proportion of CO_2_ emissions embodied in electricity rose to about 95%.

Second, significant heterogeneity in CO_2_ emissions across different manufacturing sectors was revealed. Specifically, regarding the 27 sub-sectors of manufacturing, the manufacturing of communications equipment, computers, and other electronic equipment accounts for the highest proportion of emissions, followed by the manufacturing of electrical machinery and equipment, and then the manufacturing of rubber. Considering the four categories, the emissions from high to low are electronic equipment manufacturing, equipment manufacturing and others, the raw material processing industry, and light manufacturing. In addition, in the observed years, the emissions embodied in electricity accounted for at least 65% of the total CO_2_ emissions in various manufacturing industries. In the future, saving electricity may become the most important measure to reduce the CO_2_ emissions of Shenzhen’s manufacturing. The application, dissemination, and promotion of electricity-saving technologies for production equipment in manufacturing enterprises should be strengthened. At the same time, manufacturing enterprises should be encouraged to consume renewable electricity and improve the efficiency of electricity.

Third, CO_2_ intensity, manufacturing structure, and energy structure contributed to the decline in CO_2_ emissions from Shenzhen‘s manufacturing, while economic activities led to its increase. Moreover, Shenzhen’s manufacturing emissions have gone through two significant stages, namely 2008–2012 and 2012–2020. The first stage is to rapidly reduce CO_2_ emissions due to the rapid elimination of low-end backward manufacturing industries with high CO_2_ intensity. The second stage is the period of slow CO_2_ emissions increase caused by the rapid growth of high-value-added manufacturing industries with low CO_2_ intensity. The reduction in manufacturing emissions intensity is the most important factor offsetting the increase in CO_2_ emissions from Shenzhen’s manufacturing industry activities during the two stages. This suggests that governments and manufacturing companies should continue to increase research and development and investment in energy-saving technologies. This also demonstrates that the manufacturing emission intensity targets and measures formulated by the Shenzhen municipal government are effective, and that it should continue to strengthen the control of CO_2_ intensity, especially the decrease in the intensity of electricity use. Furthermore, the Shenzhen municipal government should continue to make full use of market mechanisms such as controlled CO_2_ emission trading, the gradual reduction of emission quotas and the raising of allowance prices of the trading market. Then companies that pursue profit maximization will actively upgrade their products, develop high value-added products with higher technology, and pursue the potential of structural upgrading in the overall manufacturing industry.

Finally, the manufacturing structure and energy structure of Shenzhen have already been upgraded to a relatively low carbon level, and the marginal impact of manufacturing restructuring on CO_2_ emissions has been relatively insignificant up to now. Through continuous industrial upgrading, Shenzhen’s manufacturing industry has undergone a process from processing to manufacturing and then to creation. Previously, there were many enterprises that processed raw materials on a client’s behalf, assemble parts for those clients and then process according to the client’s samples or engage in compensatory trade. Shenzhen’s economy then basically went through labor-intensive manufacturing, such as original equipment manufacturing, before finally entering the product design and technology research and development field at the top of the value chain. Shenzhen has been striving to develop strategic emerging industries with high added value, high technology content, strong innovation, and low carbon intensity. These manufacturing industries have gradually become the pillar industries of Shenzhen, especially the manufacturing of communication equipment, computers, and other electronic equipment. Manufacturing structural optimization is one of the important determinants of Shenzhen’s manufacturing emissions. Therefore, in the future, the potential of emission reduction through the optimization of manufacturing industry structure and energy structure is expected to be gradually exhausted.

## Figures and Tables

**Table 1:** Representative studies of CO_2_ emission changes using the LMDI method.

Author	Region	Period	Mega City	Stages	Sector	Electricity Emission	Activity Effect	Structure Effect	Intensity Effect	Energy Mix Effect
Akbostanci et.al (2011) [11]	Turkish	1995–2001	× ^1^	×	Manufacturing	√	√	√	√	√
Hammond and Norman (2012) [12]	UK	1990–2007	×	√	Manufacturing	√	√	√	√−	√
Jeong and Kim (2013) [13]	Korea	1991–2009	×	×	Manufacturing	√	√+	√−	√−	√+
Roman et.al (2018) [14]	Colombia	1990–2012	×	√	All	×	√+	×	√−	√+
Mousavi et al. (2017) [15]	Iran	2003–2014	×	×	All	√	√	√	√	√
Liu et.al. (2019) [5]	China	1995–2015	×	√	Manufacturing	√	√+	√−	√−	√+
Wang and Feng (2017) [16]	China	2000–2014	×	×	All	√	√+	√+	√−	√
Wu et.al. (2016) [17]	Inner Mongolia, China	2003–2012	×	×	Industry	×	√+	√+	√−	√+
Wang et.al. (2016) [18]	Taiwan, China	2007–2013	×	×	Industry	×	×	√+	√−	√+
Feng et.al. (2019) [19]	Guangdong, China	1995–2015	×	×	All	√	√+	×	√−	√−
Gu et.al (2019) [25]	Shanghai, China	1995–2016	√	×	All	√	√+	√−	√−	√−
Kang et al. (2014) [27]	Tianjin, China	2001–2009	√	√	All	√	√+	√	√−	√
Tan et al. (2016) [28]	Chongqing, China	2000–2012	√	√	All	√	√+	√+	√−	√+
Feng et.al (2019) [24]	Zhuhai, China	2006–2016	×	×	Industry	√	√	√	√−	√
This Study	Shenzhen, China	2008–2020	√	√	Manufacturing	√	√(+)	√(−)	√(−)	√(−)

^1^ Note: “×” indicates that the document does not belong to this option; “√ ” means the item is in the document; “+” and “−” represent the positive and negative effects, respectively.

**Table 2:** Carbon dioxide emissions coefficient of various fossil fuels and electricity.

Type of Energy	Raw Coal	Crude	Gasoline	Kerosene	Diesel	Fuel Oil	LPG	Natural Gas	Electricity
Energy ID	1	2	3	4	5	6	7	8	9
Unit	t/t	t/t	t/t	t/t	t/t	t/t	t/t	t/10^4^ m^3^	t/10^4^ kWH
CO_2_ Emission Coefficient in this study	1.880	3.020	2.925	3.033	3.096	3.170	3.101	21.622	5.233
CO_2_ Emission Coefficient in IPCC	Coking coal 2.668 Anthracite 2.625 Lignite 1.202	3.101	3.186	3.153	3.186	3.127	2.985	26.928	—

Note: CO_2_ emission coefficient from IPCC (2006) was calculated through the default net calorific values (NCVs) and the default effective CO_2_ emission factor in the guidelines.

**Table 3:** Definitions of the variables.

Multipliers	Abbreviation	Meaning	Units
*C_ij_/C_j_*	CS	The structure of CO_2_ emissions of different energies	1
*C_i_/Y_j_*	CI	CO_2_ emission intensity	Ton of CO_2_ emissions per 10,000 RMB ^1^
$Y_{j}/Y$	MS	Sector structure of manufacturing	1
Y	Y	Economic activity of manufacturing	10,000 RMB

^1^ Rem Min Bi, the currency unit of China.

**Table 4:** The classification of Shenzhen’s manufacturing.

Sector	Manufacturing	Category
ID_1	Processing of Food from Agricultural Products	Light manufacturing
ID_2	Manufacturing of Foods	
ID_3	Manufacturing of Wine, Beverages and Refined Tea	
ID_4	Manufacturing of Tobacco	
ID_5	Manufacturing of Textile	
ID_6	Manufacturing of Textile Wearing Apparel, Footwear and Caps Manufacturing of Leather, Fur, Feather and Related Products	
ID_7	Processing of Timber, Manufacturing of Wood, Bamboo, Rattan, Palm Fiber & Straw Products	
ID_8	Manufacturing of Furniture	
ID_9	Manufacturing of Paper and Paper Products	
ID_10	Printing and Record Medium Reproduction	
ID_11	Manufacturing of Cultural, Educational and Sports Articles	
ID_12	Processing of Petroleum, Coking and Nuclear Fuel Processing	Raw material process industry
ID_13	Manufacturing of Raw Chemical Materials and Chemical Products	
ID_14	Manufacturing of Medicines	
ID_15	Manufacturing of Chemical Fibers	
ID_16	Manufacturing of Rubber	
ID_17	Manufacturing of Non-metallic Mineral Products	
ID_18	Smelting and Pressing of Ferrous Metals	
ID_19	Smelting and Pressing of Nonferrous Metals	
ID_20	Manufacturing of Metal Products	Equipment manufacturing and others
ID_21	Manufacturing of General-purpose Machinery	
ID_22	Manufacturing of Special-purpose Machinery	
ID_23	Manufacture of Railways Ships Aerospace and Other Transport Equipment	
ID_24	Manufacturing of Electrical Machinery and Equipment	
ID_25	Manufacturing of Measuring Instruments, Metal Products, Machinery and Equipment Repair and Other Manufacturing	
ID_26	Recycling and Disposal of Waste	
ID_27	Manufacturing of Communication Equipment, Computers and Other Electronic Equipment	Electronic equipment manufacturing

Note: All manufacturing subsectors are included. Except for ID_6, ID_16, ID_23, and ID_25, the rest of the manufacturing subsectors remain consistent with the yearbook. ID_6 is combined with “Manufacturing of Textile Wearing Apparel, Footwear, and Caps” and “Manufacturing of Leather, Fur, Feather, and Related Products”; ID_16 consists of “Manufacturing of Rubber Products” and “Manufacturing of Plastic Products”; ID_23 consists of “Manufacturing of Automobiles” and “Manufacturing of Railways Ships Aerospace and Other Transport Equipment”; ID_25 consists of “Manufacturing of Measuring Instruments”, “Manufacturing of Metal Products, Machinery and Equipment Repair”, and “Other Manufacturing”.

**Table 5:** The proportion of CO_2_ emissions embodied in electricity to total emissions in 27 manufacturing subsectors (%).

Sector	2008	2009	2010	2011	2012	2013	2014	2015	2016	2017	2018	2019	2020
ID_1	60	45	63	92	79	80	79	80	80	81	82	82	82
ID_2	66	41	57	80	66	68	71	73	75	73	69	69	71
ID_3	42	45	56	73	65	62	70	72	64	61	59	63	65
ID_4	67	73	67	90	79	70	69	72	71	72	71	75	82
ID_5	29	54	68	88	83	86	86	86	86	86	86	83	83
ID_6	61	56	64	93	89	88	90	91	91	92	92	92	95
ID_7	83	68	67	95	88	90	92	92	91	80	76	79	73
ID_8	50	49	66	95	89	90	89	89	92	92	94	91	90
ID_9	47	46	61	85	78	70	79	80	84	82	74	68	76
ID_10	40	54	68	96	91	92	92	93	93	94	95	94	95
ID_11	48	53	64	95	89	91	92	93	93	92	92	93	92
ID_12	39	53	52	96	55	53	41	42	54	46	56	63	65
ID_13	59	57	59	88	77	80	84	87	87	90	93	94	95
ID_14	46	51	69	93	77	78	78	80	76	78	78	80	80
ID_15	42	61	69	76	97	82	84	84	81	82	82	49	99
ID_16	61	65	72	94	90	90	92	92	92	93	94	95	95
ID_17	38	46	61	73	67	69	83	83	83	80	78	76	77
ID_18	29	49	67	94	84	79	76	76	80	68	62	68	63
ID_19	58	50	62	88	75	78	81	82	82	75	80	83	84
ID_20	52	55	70	91	84	85	88	86	89	91	91	91	93
ID_21	61	66	72	92	89	88	90	90	90	90	90	92	96
ID_22	68	71	79	95	94	95	95	95	95	94	96	96	98
ID_23	52	50	65	83	79	80	82	86	84	84	85	84	87
ID_24	51	57	73	95	93	94	95	96	96	97	97	96	97
ID_25	52	54	69	93	93	94	94	94	94	96	95	94	95
ID_26	-	-	61	22	97	94	97	71	88	100	100	100	100
ID_27	70	75	82	97	96	97	97	97	98	98	98	98	98

**Table 6:** Decomposition results of carbon emission changes and contributions of various driving forces.

Stage	Period	Changes in Manufacturing Carbon Emissions (10^4^ tons CO_2_)					Contributions of Various Driving Factors (%)				
		Overall Changes	CS	CI	MS	Y	Growth Rate	CS	CI	MS	Y
Stage I 2008–2012	2008~2009	−221	−4	−458	−151	392	−8.35	−0.13	−17.31	−5.71	14.80
	2009~2010	283	7	−92	38	330	11.66	0.30	−3.80	1.58	13.59
	2010~2011	−405	−17	−555	−77	243	−14.96	−0.61	−20.48	−2.83	8.96
	2011~2012	−922	−5	−1086	−14	183	−39.98	−0.22	−47.11	−0.60	7.95
Stage II 2012–2020	2012~2013	2	0	−152	11	143	0.16	0.01	−10.99	0.81	10.34
	2013~2014	50	0	−47	−26	124	3.60	−0.03	−3.42	−1.91	8.95
	2014~2015	17	0	−100	−15	133	1.22	0.00	−6.95	−1.08	9.24
	2015~2016	21	0	−93	−11	125	1.46	0.00	−6.40	−0.76	8.63
	2016~2017	53	0	−51	−32	135	3.56	0.00	−3.44	−2.16	9.16
	2017~2018	39	0	−74	−17	129	2.52	0.00	−4.84	−1.10	8.47
	2018~2019	78	0	2	13	63	4.98	0.00	0.11	0.83	4.03
	2019~2020	−103	−1	−128	−17	42	−6.29	−0.05	−7.80	−1.01	2.57
Cumulative effect	2008–2020	−1109	−19	−2835	−298	2043	−41.87	−0.71	−107.04	−11.24	77.13

## Data Availability

The energy activity data of different manufacturing sectors are from the Shenzhen Statistical Yearbook 2009~2021 published by Shenzhen Statistical Bureau. The total added value data of industry is also from the Shenzhen Statistical Yearbook 2021. All value-added data are at the 2010 price based on the industrial value-added index.
